# Assessing the Need for a Specialist Service for People with Intellectual Disabilities and Mental Health Problems Living in Israel: A Qualitative Study

**DOI:** 10.3389/fped.2013.00049

**Published:** 2013-12-19

**Authors:** Amanda Sinai, Shirli Werner, Mike Stawski

**Affiliations:** ^1^Division for Intellectual and Developmental Disabilities, National Institute of Child Health and Human Development, Ministry of Social Affairs and Social Services, Jerusalem, Israel; ^2^Paul Baerwald School of Social Work and Social Welfare, Hebrew University of Jerusalem, Jerusalem, Israel; ^3^Schneider Children’s Medical Center, Petach Tikva, Israel

**Keywords:** intellectual disability, mental health care, mental health services, qualitative, stakeholders

## Abstract

**Background:** It is well established that people with intellectual disabilities are at higher risk of developing mental illnesses. This study aimed to assess the need for a specialized service for people (children and adults) with intellectual disabilities and mental health problems living in Israel.

**Methods:** Our research question was: is there a need for a specialist mental health service for people with intellectual disabilities living in Israel and, if so, what type of service would be most appropriate? We conducted a qualitative study using semi-structured interviews with 14 major stakeholders to identify key themes in response to our research question. The data were coded and themes were identified.

**Results:** Participants were generally not satisfied with current mental health care for people with intellectual disabilities and there was a general agreement that services are in need of improvement. We identified three major themes from the data. These were: current services, future services, and ways to facilitate change.

**Conclusion:** We hope that our findings will be instrumental in shaping the ongoing debate about the best form of delivery of services to this population in Israel. Specifically, we suggest the development of a more specialized system, with the formation of multidisciplinary regional assessment and treatment units in parallel with improved relevant training for all mental health workers and the possibility of referral to specialized teams in more complex cases.

## Introduction

In line with the UN Convention on the Rights of Persons with Disabilities (1), in Israel, as in a number of other countries, there is a general trend toward integrating people with intellectual disabilities within their communities. This has led to initiatives in Israel such as, for example, developing accessible playgrounds for children of all abilities (2), including children with intellectual disabilities within regular school classes (3), and including adolescents with intellectual disabilities within regular youth groups (4). Also, an annual delegation of Israelis with intellectual disabilities now travels to Poland on a Holocaust remembrance program alongside others who do not have intellectual disabilities (5). Despite this positive trend, people with intellectual disabilities are often excluded from many services, including health, education, and community facilities.

This study focused specifically on people with a dual diagnosis of both intellectual disability and mental illness. It is well established that people with intellectual disabilities have higher rates of mental illness (6). Assessing and diagnosing a person with intellectual disabilities and mental health problems is usually more complex than in the general population, due to factors such as communication difficulties and/or comorbid physical health problems. Therefore, frequently people with dual diagnosis are more likely to be excluded from various services, and in particular health services, which are the focus of this manuscript. Given their more complex needs, one would expect people with intellectual disabilities to be given more support by mainstream services. However, paradoxically, it may be more difficult for them to access mainstream services and it may also be more difficult for mainstream services to tailor their management to them. This has sometimes led to healthcare inequalities for people with intellectual disabilities (7).

Some countries, such as England, have specialist mental health services for people with intellectual disabilities (8). In some other countries, such as Australia (9), people with intellectual disabilities are managed by general psychiatric services, with access to a specialist service available for education and consultation. Israel has a mental health disability law which provides access to rehabilitation services (including supported employment and case management) for people with mental health difficulties (10); however, it is not clear how easy it is for people with intellectual disability and mental illness to access these services and how well they are tailored to their needs.

The psychiatric/psychological treatment of people with intellectual disabilities and mental health problems living in the community in Israel are currently managed by general (child/adolescent or adult) mental health services. Those living in group homes or larger residential facilities often have access to a visiting psychiatrist. There is no mandatory specialist training for psychiatrists who work with people with intellectual disabilities, and their contact with other professionals working in the same field may be limited. Furthermore, it has been previously pointed out that current mental health services for people with intellectual disabilities living in Israel are in need of improvement (11).

The need for good mental health care for this population is highlighted by the fact that around 50% of people living in residential care in Israel receive psychotropic medication (12). However, a recent study among public sector psychiatrists in Israel has found that most psychiatrists report limited training and inadequate knowledge in the diagnosis and treatment of people with intellectual disabilities. Interestingly, those psychiatrists with fewer years of experience and those working with children and adolescents reported more positive attitudes toward this population than those with more years of experience or those working with adults (13). Also, it has been shown that psychiatrists with lower self – reported levels of knowledge about people with intellectual disabilities report lower levels of satisfaction when treating people with intellectual disabilities (13).

Although this topic is an important issue and continues to be discussed both amongst governmental and non-governmental organizations in Israel, it has not been assessed using a qualitative perspective in Israel and from the perspective of major stakeholders in the field. Thus, the aim of this study was to assess the need for a specialized service for people (children and adults) with intellectual disabilities and mental health problems living in Israel.

Our research question was: is there a need for a specialist mental health service for people with intellectual disabilities living in Israel and, if so, what type of service would be most appropriate?

## Materials and Methods

We conducted a qualitative study using a semi-structured interview in order to identify key themes in response to our research question. We invited major stakeholders to participate in the study. Potential stakeholders to interview were identified by one of the authors (MS), who has experience working with people with intellectual disability living in Israel, or at the suggestion of participants (using a snow-ball technique). Those invited to participate, included individuals working within governmental and non-governmental organizations, including the Ministry of Health, the Ministry of Social Affairs, service user groups, and individual clinicians working with people with intellectual disabilities. This allowed us to examine the perspectives of policy makers, of people working as policy campaigners and of individuals working directly with people with intellectual disability and mental health problems.

The interview guide was discussed and drawn up by two of the researchers (AS and MS), both psychiatrists with experience in intellectual disability. It was based on topics that were thought to be most relevant to the research question. The interview guide was piloted on two participants and slightly amended following this (two of the questions were combined). This resulted in a total of one introductory section and four additional probing sections. See Box 1 for further details. Participants also had the option of adding any further comments or suggestions at the end of the interview.

Box 1 **Interview guide.**

Introductory section: what is your experience working with people with intellectual disabilities and mental health problems in Israel? Please describe your experience. Do you think people with intellectual disabilities have higher rates of mental health problems/behavioral disturbance than other people?

Are you satisfied with current mental health services for people with intellectual disabilities in Israel, including inpatient and outpatient services? Are there any areas of weakness in current mental health services for people with intellectual disabilities? If so, what are they?

What should be done (both in an ideal world and in practice) to improve mental health services for people with intellectual disabilities?

Is there a need for specialist services for people with intellectual disabilities and mental health problems? Should all mental health workers be trained to support people with intellectual disabilities?

What is your opinion about the proposed reform of mental health services in Israel? Should it comment on mental health in people with intellectual disabilities and, if so, what should it say?

Interviews were conducted by the primary researcher (AS) with each of the participants at a time and place that was convenient for the participant. Interviews were conducted in English, as this was the native language of the researcher. Participants were given the option of having a person to help interpret if required. Participants were sent a further email after the interview and were given a contact email address if they had any further questions or comments.

Fourteen stakeholders were interviewed. They came from a variety of professional backgrounds, including working in the Ministry of Social Affairs, Ministry of Health, not-for-profit organizations, and psychiatrists working with people with intellectual disabilities. One participant, as well as having a professional role, was also the parent of an adult with intellectual disability.

Interviews were conducted between May and November 2011; they ranged in length from 7 to 46 min. All participants were comfortable speaking in English and an interpreter was not required for any of the interviews.

Data were recorded using a computerized recording package (Audacity 1.3). Data from the pilot phase were included in the analysis. Data were transcribed and transcriptions checked and then coded by the primary author (AS). Themes were then identified from these codes.

The study was approved by the Ethics Committee of the School of Social Work within the Hebrew University in Jerusalem.

## Results

We identified three major themes from the data. These were: current services, future services, and ways to facilitate change.

### Current services

All 14 participants were dissatisfied with current mental health care for people with intellectual disabilities. Of these, two participants were not satisfied with mental health care in general in Israel.

Sub-themes focused on dissatisfaction with services and contributing factors.

1. Dissatisfaction with services was attributed to two main reasons.
  - Scarcity of services – a common sub-theme was the scarcity of services. As one participant said, “I think that we don’t have enough professionals who are dealing specifically with this population.” This sub-theme included comments that only a few professionals with a special interest were working in the field and that long waiting lists means that patients often need to be seen privately. Participants also mentioned that psychiatrists often work alone (i.e., without a multidisciplinary team) and during limited hours. One participant highlighted that there are currently no specialized inpatient units for children with mental health problems and intellectual disability in Israel.
  - Variability of services – participants highlighted differences between services received by individuals living in residential care, who often have access to a private psychiatrist, and those who live in the community, who are expected to access general community mental health care, but often have difficulty doing so. Also mentioned was that services are not standardized and vary according to geographical area. Other comments included that psychiatrists rely mainly on a “medical model” of practice, while frequently an additional social perspective may be more complete and appropriate in such cases. Furthermore, participants commented that medications may not always be reviewed and that sometimes psychiatric medications may be started without a detailed assessment.
2. Contributing factors.

Participants commented on various factors that may contribute to current service inadequacies.

- Specific challenges in working with people with intellectual disabilities – this sub-theme included challenges in communication and difficulties in the assessment process. Comments included that assessment is often more complex and can take more time, as it may require gathering a detailed history from a number of different sources, amongst other things. Problems associated with diagnostic overshadowing (ascribing a presenting problem to a person’s intellectual disability and missing an underlying health issue) were commonly mentioned. As one participant said, “It’s not easy to diagnose mental disorder in (the) intellectual disability population. I think … (there is still some diagnostic) overshadowing.”
- Attitudes of professionals – many participants mentioned the attitudes of professionals as contributing to the current situation. Some of them referenced previous research with which they were familiar that supported the existence of negative attitudes (14). Comments included that professionals may not want to work with this group of people and that it can be complicated work.
- Organizational difficulties – participants commented on many organizational difficulties that may lead to difficulties accessing appropriate services. This included comments that mental health services are weighted toward people living in institutions (who often have access to in-house psychiatrists) rather than community services. Also mentioned was that access to services is through diagnosis rather than need, which may be problematic if it is difficult to make a clear diagnosis, or if a patient has a diagnosis but a greater need due to their intellectual disability. Also discussed was that service developments require liaison between Governmental Ministries and non-governmental organizations, but that it may be difficult to determine how to share the responsibility between them, and patients often get left in the middle.

### Future services

Participants commented on what they hoped services would look like in the future.

1. Specialized versus general services – there was no consensus as to whether specialized or general services were ideal, and throughout the course of a number of interviews, participants changed their minds, or had to pause to think about things. The majority of participants did think that there should be specialized services for people with intellectual disabilities and mental health problems, although some suggested a partially specialized system with general mental health teams managing people with intellectual disabilities on a daily basis and more specialized teams providing advice or consultation. Some participants suggested the development of residential assessment and treatment centers, which could also act as a base for the specialized teams. Interestingly, there was not necessarily a link between seeing people with intellectual disabilities as a special group with special needs, and being in favor of specialized services. For example, a number of participants talked about people with intellectual disabilities as having the same rights as other people and then went on to suggest specialized services.
  Three participants were not supportive of specialist services, but the reasoning behind this position differed between them. One participant believed that specialized services would carry high financial costs. Another participant believed that if a philosophy of specialized services were to be adopted there would be a great demand for additional specialist services for other population groups. The third participant expressed concerns regarding stigmatization and exclusion which may arise when individuals are supported in separate services. However, all three participants were in favor of increased training for professionals in the field.
  Other participants also commented on the practicalities of economic factors and the likelihood that there are a number of different patient groups advocating for specialist services, which may limit the development of specialist services for this group. However, one participant highlighted the fact that in child and adolescent mental health, there are specialist services for a number of different groups (e.g., for children or adolescents with anxiety problems or ADHD), but not for people with intellectual disabilities.
2. Multidisciplinary approach – participants commented on use of a multidisciplinary or systems approach, and the value of this, as well as the importance of professionals making connections with other individuals and organizations working with the individual. Some participants recommended a needs-based approach and treating people according to their need. Examples included allocating more time during assessment and using the appropriate members of the multidisciplinary team. One participant highlighted the importance of supporting families. Two participants described the importance of a person-centered approach. One participant suggested that it may be more appropriate for a person with long-standing behavioral problems to be managed using a social approach. As one participant said:
  “The team must be multi-disciplinary. You, you need to make (many) more connections around the treatment and around the recommendation with all kinds of services and systems in the community. So you must work in a system approach, much more than we see today, it’s not only the child and the adult and family but also the professionals that work directly and also professionals that work not directly but are involved in the case.”
3. Service models – apart from improving current general services, a number of specific service models were suggested. These are shown in Box 2.

Box 2 **Future services: suggested service models.**

Improvements in general training for all mental health workers and subsequent referral to specialized psychiatrists in more complex cases.

The employment of full time specialized psychiatrists in intellectual disability and the development of a specialist network of psychiatrists.

Development of specialized multidisciplinary outpatient community services, which can manage people in crisis and then provide support to local teams.

Health Funds (Kupot Cholim) to manage community clinics (including mental health services) in institutions.

Development of regional assessment and treatment units, with long stay beds for people with more chronic problems and shorter stay assessment and treatment beds.

Development of a specialized child and adolescent inpatient unit.

### Ways to facilitate change

Participants made several different recommendations on how the current system could be changed and improved.

1. Education and training – this was one of the topics most frequently discussed and although teaching was a specific question in the interviews, many participants mentioned teaching or training before being asked specifically about it. Some participants noted that although previously training about mental health problems in people with intellectual disabilities had been organized, attendance by psychiatrists was poor. A number of participants thought that the best option would be basic training for all psychiatrists (and some participants suggested also for medical students, psychiatry trainees, all doctors, and mental health workers) as well as more specialized training for those that are interested in specializing in the field. Some suggested the need to develop a unit for training and supervision of professionals. It was also suggested that training should be compulsory for those psychiatrists who were working in the institutions/residential centers. Topics suggested included clinical teaching, assessment, diversity training, communication skills and teaching via hands-on experience, and face to face contact with people with intellectual disabilities. One participant suggested that protocols should be developed to aid psychiatrists in their management plans if they are unfamiliar with working with people with intellectual disabilities. Another suggestion was that psychiatrists in training should be supervised to review residents at a residential home or institution on a regular basis. As one participant said: “we really need to open a unit of studying and supervision (with) people who have experience (in intellectual disability and mental health problems) and are at a high level … professional level.”
2. Inter-organizational working – a number of participants commented on the particular importance of governmental ministries working together, in order to avoid people being left between the gaps. It was suggested that this would involve further communication and collaboration between The Ministry of Health, Ministry of Social Affairs, and Ministry of Education. Also highlighted was the link between governmental and non-governmental organizations. As one participant said, “It should be a collaboration of social services, education, and health services from a very early age and through, you know, high school and adult life, because if they collaborate and will be trained and have good communication, then we can solve some of the problems.”
3. Mental health reforms – the Government of Israel continues to progress in moving the mandate for provision of mental health services from the government to the health funds (Kupot Cholim) (10). This movement has continued to progress since the time that these interviews were conducted. Participants were divided in their opinion as to whether the reform will be better for this group. One participant commented that at present, services are minimal, so any change could only be an improvement. Some participants were concerned that if the reform did not highlight people with intellectual disability as a group with different/special needs, they will receive a standard treatment package and not one that is specifically tailored to their needs. Again, it was not necessarily the same participants who thought people with intellectual disabilities should have specialist mental health services and those who thought they should be mentioned as a specific group in the mental health reforms.

Encouragingly, many participants described a sense of responsibility and ownership regarding the development of mental health services for this group. As one participant said “the gap … the gap is not, is not so wide, you just have to be more open to learn it … psychologically minded, and this is my challenge. To make it … make this process.”

## Discussion

This study aimed to assess the need for a specialized service for people with intellectual disabilities and mental health problems living in Israel from the perspective of various governmental and non-governmental stakeholders in the field. In general, participants were not satisfied with current mental health care for people with intellectual disabilities and there was a general agreement that services are in need of improvement. There were differences of opinion regarding development of specialized services for people with intellectual disabilities, although the majority of participants highlighted the importance of either specialized or needs-based care.

Stakeholders displayed a sense of ownership and were keen to improve mental health services for this population, but also highlighted difficulties, due to individual and organizational factors. Most participants highlighted training as an important step in improving mental health services for this population. Some participants suggested developing a network of professionals. Others suggested ways to improve services, which included inter-organizational working and using the upcoming mental health services reform to facilitate change.

### Strengths and limitations

Several limitations should be kept in mind when considering the results of this study. First, we deliberately selected a sample of participants who were involved in the field, and therefore this is not a generalizable sample, which needs to be taken into account when examining the data. We also acknowledge the researcher’s influence when collecting and analyzing data (in particular, the fact that the primary researcher was a UK trainee in Intellectual Disability Psychiatry) which may have influenced the participants’ answers somewhat and may have also influenced the analysis of the data. This is an expected phenomenon in qualitative research and is an important part of data collection and analysis, and we acknowledge this when interpreting and reporting our data. Nevertheless, this study explores an important topic in detail. By using a qualitative approach, we were able to explore several different opinions and viewpoints and put forward a framework for further thinking and research on the topic.

### Findings in the context of practice elsewhere

Findings from this study reflect opinions in other parts of the world (15, 16). There continues to be ongoing debate regarding inclusion within generalized services versus provision of specialized services, and this, combined with geographical, economic, governmental, and other factors means that across the world, mental health services for this population vary. This may be problematic in terms of achieving the requirements called upon in the UN Convention on the Rights of Persons with Disabilities. Specifically, this convention highlights the right of people with disabilities to have access to the same range and quality of healthcare (including access to mental health services) as other people (1). Given that mental health services vary between countries, it is possible that people are not being fully supported in achieving their rights.

Although this study aimed to assess the need for a specialized mental health service for people with intellectual disability living in Israel, it may be that in other countries there is also a need to consider mental health service provision for this group. We hope that our research will help to add to the ongoing debate about how to provide the best mental health care for people with intellectual disability not just in Israel but also in other parts of the world.

One sub-theme was scarcity of services, including comments that psychiatrists often work alone. It is important to remember the dangers of professional isolation, which were evident, for instance, in the recent Winterbourne View case in England which involved severe physical and emotional abuse (17), and try to find solutions to this.

Another sub-theme was variability of services, which included comments regarding medication prescribing and medication review. Doctors treating patients with intellectual disability should conduct a detailed assessment of the individual’s emotional and behavioral disturbance (18), assessing physical and mental health as well as psychological and social factors before considering psychotropic medication. If psychotropic medication is prescribed, this should be regularly reviewed, with careful consideration and documentation of the risks and benefits.

A multidisciplinary approach was another sub-theme in the theme of future services. In England, specialty intellectual disability teams are often multidisciplinary and can include Psychiatrists, Psychologists, Speech and Language Therapists, Occupational Therapists, Physiotherapists, and Nurses. They may also be integrated with Social Services. This works alongside a bio-psycho-social approach, where biological, psychological, and social factors are considered in assessment and management of mental health problems in people with intellectual disability (19, 20).

## Conclusion

We call on stakeholders to consider how best to develop mental health services for people with intellectual disabilities living in Israel. An appropriate initial development would be a two-tier system of improving general training for all mental health workers with subsequent referral to specialized mental health teams in more complex cases. This could be further developed into a more specialized system, with the formation of multidisciplinary regional assessment and treatment units, which could incorporate outpatient community services (which could manage people in crisis and then provide support to local teams), and inpatient assessment and treatment beds.

We hope that our findings will be instrumental in shaping the ongoing debate about the best form of delivery of services to this needy population. In particular, relatively simple yet effective starting points include the importance of developing education and training for mental health professionals, and developing a network for specialists. The concept of multidisciplinary team working and person-centered planning is also an important way forward in supporting this population.

## Author Contributions

Amanda Sinai developed the research question and interview guide, conducted the interviews, analyzed the data, and drafted the manuscript. Shirli Werner aided in drafting the interview guide, analyzed the data, and drafted the manuscript. Mike Stawski developed the research question and interview guide, recruited participants, drafted the interview guide, and drafted the manuscript.

## Conflict of Interest Statement

The authors declare that the research was conducted in the absence of any commercial or financial relationships that could be construed as a potential conflict of interest.
